# Hydration Forces Dominate Surface Charge Dependent Lipid Bilayer Interactions under Physiological Conditions

**DOI:** 10.1021/acs.jpclett.1c02572

**Published:** 2021-09-17

**Authors:** Valentina Wieser, Laura L. E. Mears, Robert D. Barker, Hsiu-Wei Cheng, Markus Valtiner

**Affiliations:** † Institute for Applied Physics, Vienna University of Technology, Wiedner Hauptstrasse 8-10, A-1040 Vienna, Austria; ‡ School of Physical Sciences, University of Kent, Canterbury CT2 7NZ, United Kingdom

## Abstract

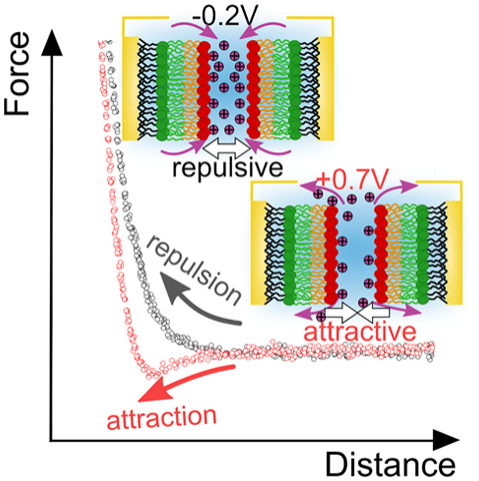

Lipid bilayer interactions are essential to a vast range of biological functions, such as intracellular transport mechanisms. Surface charging mediated by concentration dependent ion adsorption and desorption on lipid headgroups alters electric double layers as well as van der Waals and steric hydration forces of interacting bilayers. Here, we directly measure bilayer interactions during charge modulation in a symmetrically polarized electrochemical three-mirror interferometer surface forces apparatus. We quantify polarization and concentration dependent hydration and electric double layer forces due to cation adsorption/desorption. Our results demonstrate that exponential hydration layer interactions effectively describe surface potential dependent surface forces due to cation adsorption at high salt concentrations. Hence, electric double layers of lipid bilayers are exclusively dominated by inner Helmholtz charge regulation under physiological conditions. These results are important for rationalizing bilayer behavior under physiological conditions, where charge and concentration modulation may act as biological triggers for function and signaling.

Cell membranes are naturally surrounded by physiological salt solution (150–300 mM), and the interaction of ions with the lipid bilayer membrane plays a fundamental role in steering biological processes. Cation–surface interactions mediate signaling mechanisms as well as transport mechanisms driven by potential gradients across membranes, and they may contribute to the general stability of bilayers.^1,2^

The lipid headgroups can have characteristic affinities for specific ions, which adsorb on the bilayer and affect the functionality via charge regulation mechanisms.^3^ Molecular dynamics studies on monovalent salts suggest that Na^+^ has a weak affinity for adsorbing on phosphatidylcholine headgroups.^4,5^ However, AFM and ζ-potential studies of zwitterionic lipids have shown that an increasing cation concentration leads to significant ion adsorption within the inner Helmholtz layer. This contributes to a significant charging of these naturally neutral surfaces and hence results in an increase of the ζ-potential.^1^ In addition, adsorption of ions due to charge neutrality conditions on the lipid headgroups increases the hydration layer thickness as ions are surrounded by a specific hydration shell.^2^ This also leads to repulsive steric hydration forces, which depend on the specific ion adsorption.^6,7^ Specifically, surface forces apparatus (SFA) studies showed significant hydration repulsion during bilayer interactions.^8−11^

In particular, upon close approach (to within a few hydrated ion radii) the expulsion of the hydration shell and the partial dehydration of headgroups result in short-range repulsive forces during bilayer–bilayer interactions^12^ as well as during lipid adsorption on solid substrates which mediates the formation of technologically relevant bilayer coatings and biomaterials for e.g. biosensors or biomedical devices.^13,14^

The hydration structure of bilayers is further very sensitive to modulations of the surface charge in general. This is particularly important in membranes containing charged headgroups as well as during electrochemical modulation of bilayers in applications such as biosensing.^15−17^ However, no *in situ* data are available about the electric double layer structure in response to a change of the surface charge.

Here, we used an electrochemical SFA^18−20^ to study the effect of charge modulation on the ion adsorption and desorption on membranes and their interaction profiles.

Figure 1a shows a schematic of the experimental SFA setting (see Supporting Information section S2 and caption for details). Briefly, as shown in Figure 1b, two gold surfaces, set up as a three-mirror force balance,^19,21^ are modified by a recently developed^22^ system of symmetric tethered bilayer lipid membranes (tBLMs) with phosphatidylcholine (PC) headgroups on electrochemically polarizable gold substrates.

**Figure 1:**
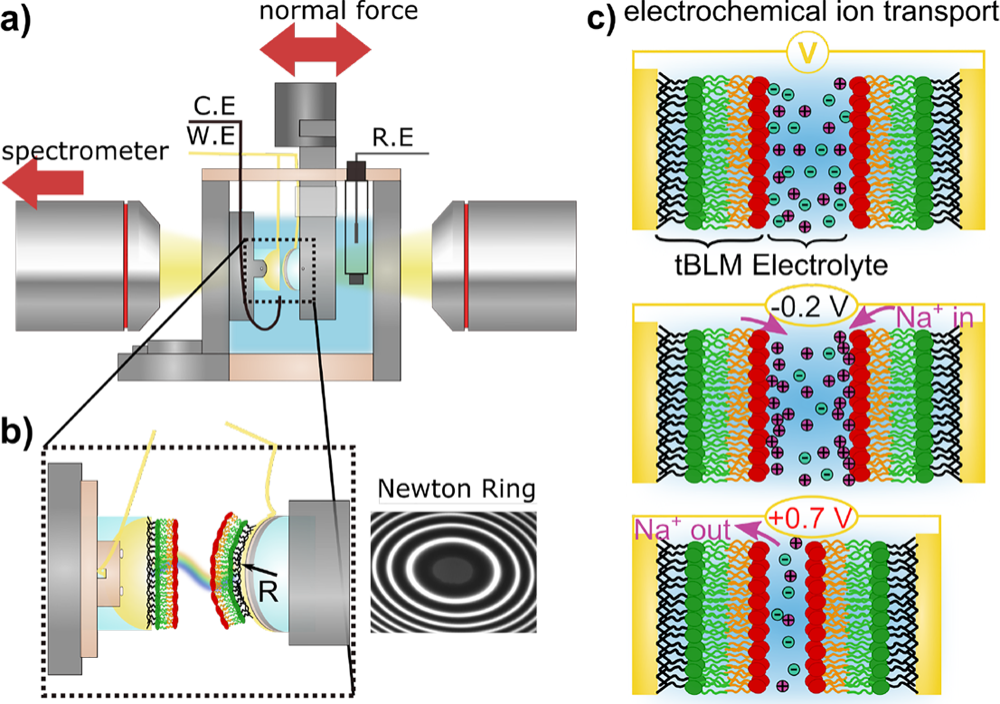
Instrumental setup of the symmetric electrochemical three-mirror interferometer surface forces apparatus. (a) Schematic of the electrochemical SFA cell with electrode arrangement. (b) Enlarged schematic of the cross-cylindrical surface arrangement with bilayer functionalization and wire connections as well as the interferometric Newton ring pattern for separation distance analysis. (c) Schematic of bilayer functionalized surfaces showing charging behavior upon polarization at negative and positive potentials, with cations adsorbing strongly at negative potential, and vice versa.

As indicated in Figure 1c, this symmetric setup allows a simultaneous and equal polarization of two apposing bilayer surfaces. Upon application of positive or negative electrochemical potentials, cations can desorb or adsorb to the surfaces, respectively.

Measuring force versus distance characteristics provides a means to directly characterize hydration and electric double layer structure modulations as a function of the applied electrochemical and hence established surface potentials.

Figure 2 compares force–distance (*F*–*D*) measurements between tBLMs in Milli-Q water and 1 mM as well as 150 mM NaCl concentration and applied potentials of −0.2 V (gray/black) and +0.7 V (light red/dark red), where *D*_0_ = 0 denotes the minimal separation distance when the two bilayers are in contact.

**Figure 2:**
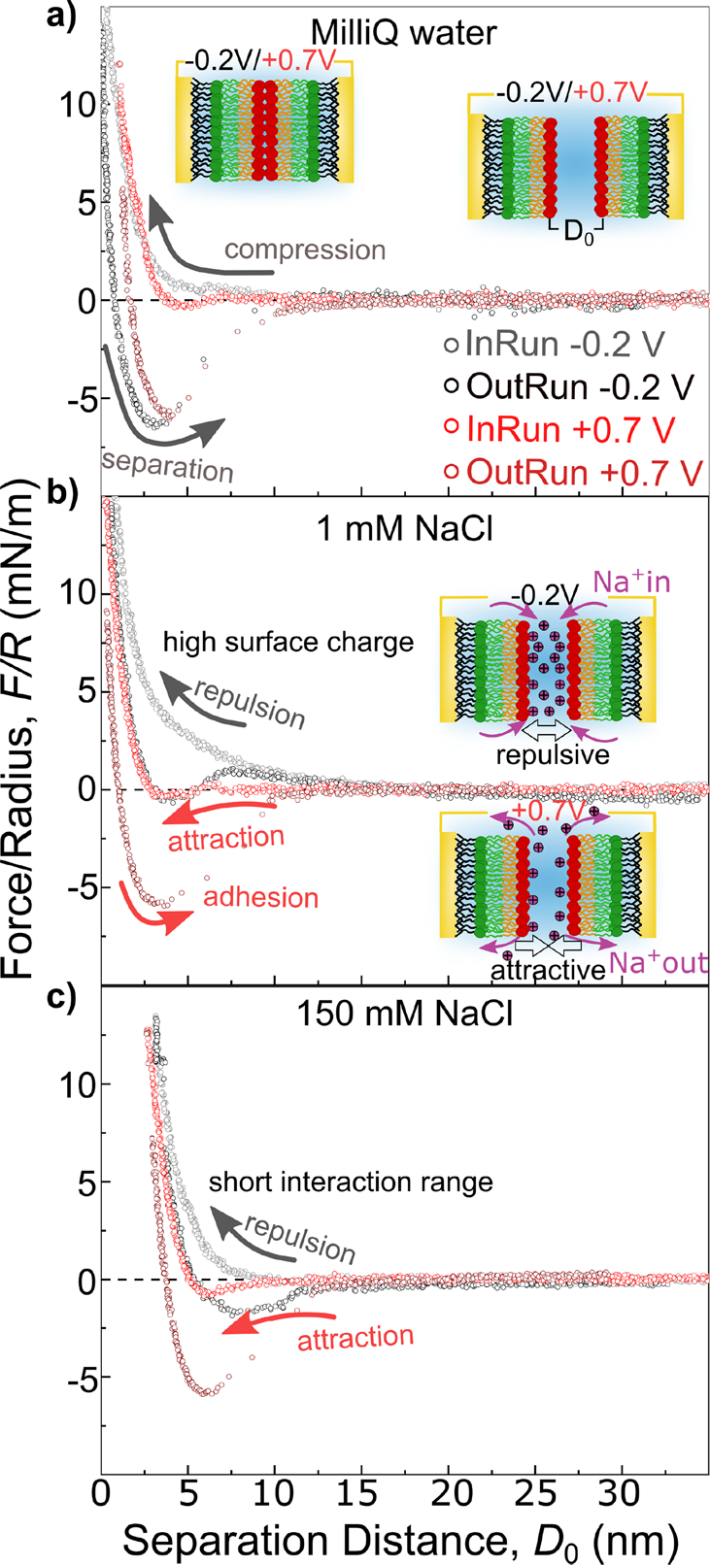
Force versus distance characteristics during approach and separation recorded between two identical bilayers at −0.2 and +0.7 V for increasing NaCl solution concentration. (a) Milli-Q water, at −0.2 V during compression (gray) and separation (black) and at +0.7 V (compression = light red and separation = dark red). (b) 1 mM NaCl concentration (gray: −0.2 V; red: +0.7 V). (c) 150 mM NaCl (gray: −0.2 V; red: +0.7 V).

Figure 2a depicts measurements in Milli-Q water showing only a minor polarization dependent trend of the bilayer interaction. The *F*–*D* characteristics during approach indicate a short-range exponential repulsion below 5–10 nm at both potentials. At positive polarization a clear attractive component lowers the repulsive force at *D*_0_ = 5 nm. This is in line with a variation of the surface charge and the water structuring at the interface. Upon separation we measured a similar adhesion force *F*_adh_ ∼ 6 mN/m for both −0.2 V (black) and +0.7 V (dark red). This is indicative of a van der Waals dominated minimum in the absence of ion adsorption.

As shown in Figure 2b, introducing ions into the system results in a considerably more pronounced polarization dependent interaction with a clear repulsive behavior at negative potentials and a pronounced attractive profile, compared to Milli-Q water, at positive potential.

In detail, the *F*–*D* curve recorded at −0.2 V (gray curve) indicates a long-range repulsion during compression and virtually no adhesion upon separation. In contrast, at +0.7 V the compression curve (light red) shows an initial repulsion followed by an attractive minimum at *D*_0_ = 4–5 nm. Further compression of the bilayers results in a hydration repulsion. During separation the system exhibits an adhesive minimum of *F*_adh_ = −6.5 mN/m (dark red).

Increasing the ion concentration toward physiological conditions at 150 mM NaCl in Figure 2c again indicates polarization dependent switching between repulsive and attractive characteristics at −0.2 and +0.7 V, respectively. However, a decrease in the repulsion range is evident for both polarizations, congruous with a decrease of the Debye length in high salt concentrations. In addition, a significant outward shift of the *F*–*D* profile of ∼3.5 nm is evident. This is consistent with a swelling of the DOPC headgroup in high salt concentration due to ion adsorption and water structuring at the interface. This assertion is also supported by neutron reflectometry data from the DPhyTL tethered support layer in Figure S1, which indicates no change in the fringes observed from the tethered layer on the gold in D_2_O. For neutrons the deuterated solvent offers the greatest contrast to the hydrogenated thin film. If the solvent were to have swollen the DPhyTL layer, the spacing between the fringes would decrease, owing to the thickness increase, which is not observed. The technique is sensitive to changes in layer thickness on the nanometer to angstrom scale.^23^ Therefore, the outward shift observed in the SFA data with the addition of salt can be assigned exclusively to headgroup hydration driven effects.

Taking into account the symmetry of the system, about 1.7 nm thickness increase can be assigned to each surface. Considering the hydrated Na^+^-ion diameter (first hydration shell) of about 8 Å, this thickness is consistent with an inner Helmholtz layer of adsorbed and structured water as well as ions at the interface.

The observed data can be fit well with an extended DLVO model, described in detail in Supporting Information S5. Briefly, the model assumes a linear superposition of van der Waals (vdW), diffuse electric double layer forces (EDL, Gouy–Chapman), and short-range exponential hydration interactions.^9,24^

Figures 3a–d show an enlarged view of the bilayer *F*–*D* compression curve including the fitted total interaction force profiles (black) with the extended DLVO model (see Supporting Information S5) and the separately plotted individual contributions of vdW (green), hydration (blue), and EDL (purple) interactions. The fitting parameters are listed in Table 1. The behavior of the force profiles indicates a stark variation of the nature of the potential dependent force modulation as follows;

**Figure 3:**
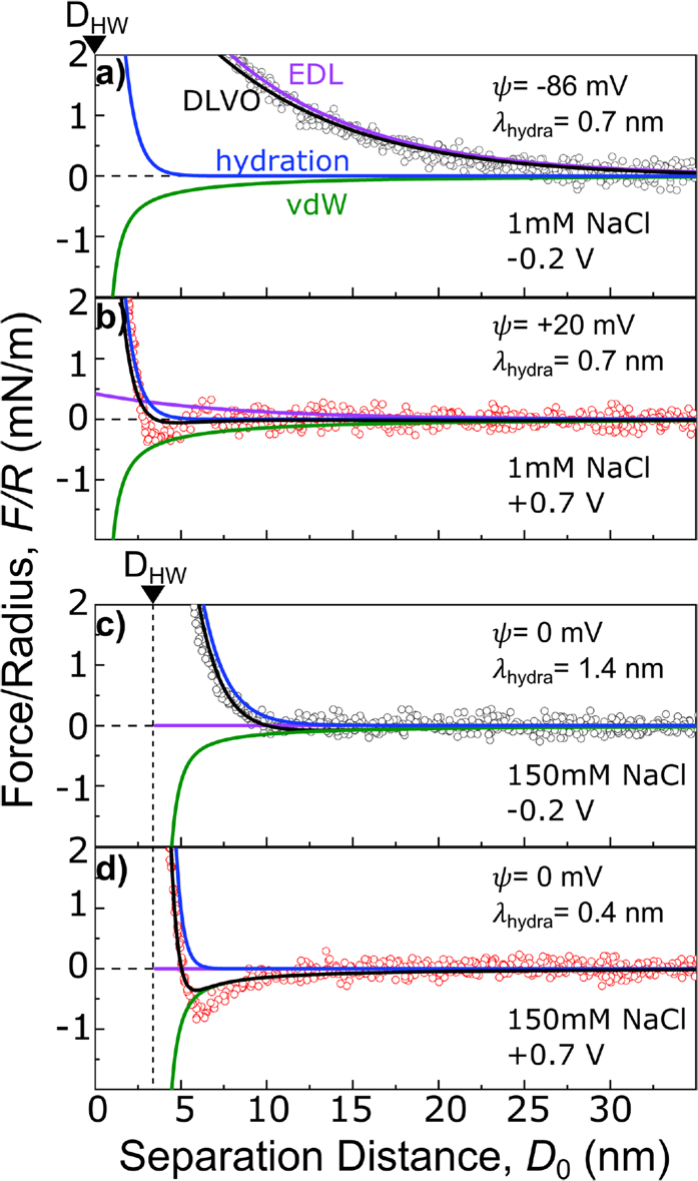
Extended DLVO fitting for approach characteristics recorded in 1 mM (a, b) and 150 mM NaCl concentration (c, d) for polarization at −0.2 V (gray) and +0.7 V (red). The solid black lines show the overall fitted interaction profile combining all three force terms in the framework of a hydration force extended DLVO model. Solid purple lines correspond to fitted electric double layer repulsion terms, solid green lines depict the fitted vdW forces, and the blue lines are fitted to the acting steric hydration forces. *D*_HW_ indicates the minimum distance at which the shown forces act.

**Table 1:** Fitting Parameters for DLVO Fit in Figure 3

*c* [mM]	*V* [mV]	κ [nm^–1^]	ψ [mV]	*A*_H,bilayer_ [10^–20^ J]	*A*_H,Au_ [10^–19^ J]	*A*_H,Au–medium_ [10^–20^ J]	*D*_HW_ [nm]	λ_hydra_ [nm]	*W*_0_ [J/m^2^]	*D*_Au_ [nm]
1	–0.2	0.125	–86	1	3	5	0	0.7	0.025	–8
1	+0.7	0.125	+20	1	3	5	0	0.7	0.025	–8
150	–0.2	1.25	0	1	3	5	3.5	1.4	0.015	–8
150	+0.7	1.25	0	1	3	5	3.5	0.4	0.042	–8

First, at low concentrations, the *F*–*D* curve at −0.2 V polarization (Figure 3a) fits well to the electric double layer repulsion for symmetric surface potentials of ψ = −86 mV.

From the DLVO fit (black) it is evident that the EDL repulsion dominates the force profile. Switching the polarization to +0.7 V in Figure 3b results in a strong decrease of the long-range EDL repulsion with a surface potential of ψ = +20 mV. In addition, at short separations the *F*–*D* curve follows a hydration repulsion for separation distances *D*_0_ < 4 nm. It is interesting to note that the only varying parameter for fitting the data at both potentials is the EDL potential, while all other parameters are constant.

Second, in 150 mM solution the compression profile for −0.2 V polarization in Figure 3c shows that the repulsive interaction has a shorter range, as expected for a lower Debye length of 0.8 nm. However, the measured exponential decay of 1.4 nm can be fit well with a steric hydration related repulsion and outward shifted vdW interactions for the bilayer contribution. The outward shift corresponds to the measured swelling of the bilayer (i.e., *D*_HW_ shift) due to ion adsorption. Interestingly, this can be well described by an effective shift of the hard wall of the lipid/lipid van der Waals contribution and the corresponding planes of origin for the hydration and electric double layer planes. This suggests that the Hamaker constant of the hydrated bilayer does not vary significantly compared to the lower hydration state, although this could be modeled by more complicated approaches.^25^

Switching to +0.7 V in Figure 3d results in an attractive vdW dominated minimum, which is again shifted outward by swelling of the hydrated bilayer (*D*_HW_). In contrast to the 1 mM case, solely a change of the hydration parameters can explain the potential dependent force modulation. In detail (see again Table 1) the hydration decay length decreases from λ_hydra_ = 1.4 nm to λ_hydra_ = 0.4 nm. A change of λ_hydra_ and the corresponding prefactor *W*_0_ is necessary to model the measured behavior. With regard to this short-range compression, Helfrich undulations were also tested,^22,26^ but a 1/*D*^2^ decay does not fit the observed remaining compression. The short exponential hydration length fits well, and in the absence of ions it seems consistent with an oriented water structure formation at the interfaces of the uncharged surface.

In summary, this suggests that charge screening of bilayers at close to physiologic concentrations is dominated by steric hydration effects, which are essentially inner electric double layer effects. The Gouy–Chapman model clearly breaks down under physiological conditions, and the extended DLVO model simplifies to a linear superposition of potential dependent steric hydration forces and van der Waals forces. In contrast, at low concentrations the extended model is necessary to describe the overall potential dependent behavior. At low surface charging steric hydration forces dominate, while at high surface charging the Gouy–Chapman repulsion dominates.

Hence, it is now clear that steric hydration effects dominate the potential dependent behavior at high concentrations. These effects may include both steric repulsions due to ion adsorptions and ion adsorption facilitated protrusion forces. That is, ion adsorption can weaken the interfacial energy and therefore increase the protrusion decay length.^27,28^ However, to further dissect the individual steric hydration force effects would require a detailed investigation beyond the scope of this work using both a multitechnique approach and temperature dependent measurements.

It is now interesting to further compare dynamic polarization changes during *F*–*D* measurements to equilibrium conditions of the bilayer as discussed so far. Therefore, Figure 4a,b shows *F*–*D* curves with a transient state, when the polarization is switched shortly before maximum compression of the bilayers, in 150 mM NaCl.

**Figure 4:**
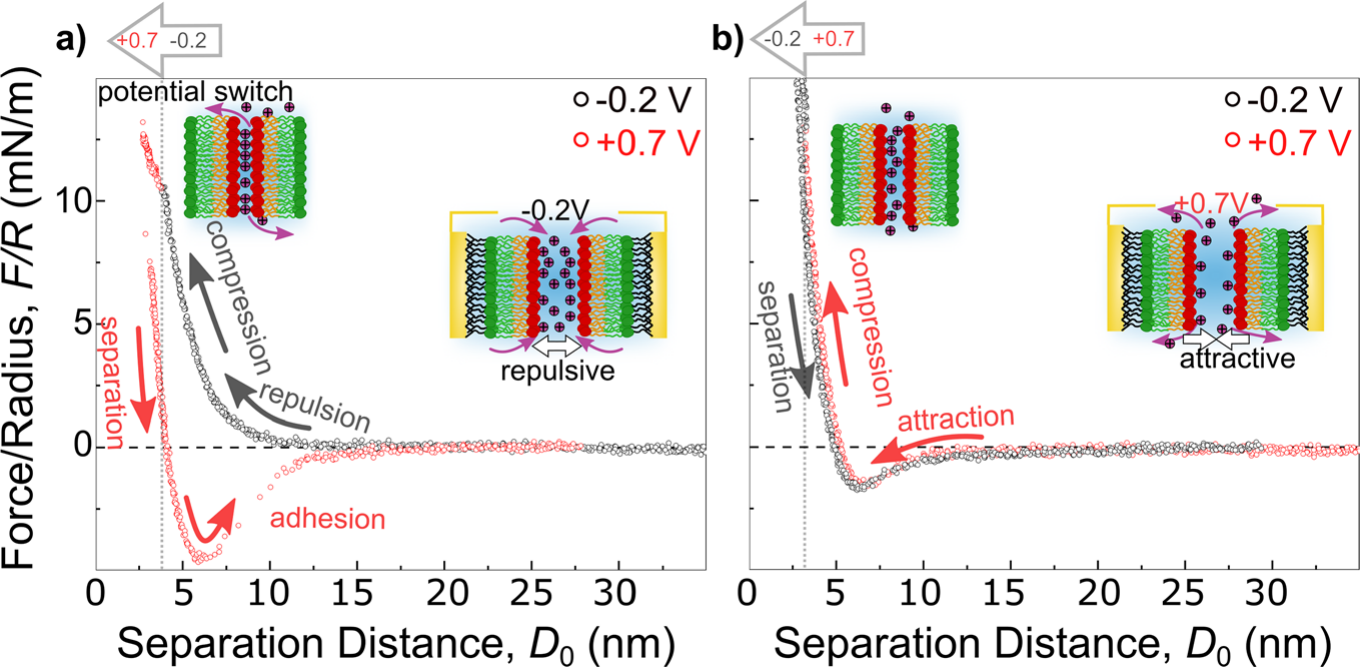
*F*–*D* measurements in 150 mM NaCl solution with dynamic polarization switch from (a) −0.2 V (black) to +0.7 V (red) during compression and from (b) +0.7 V to −0.2 V. The switch from −0.2 V to +0.7 V, indicated by a change in color from black to red, and by the arrow on top, shows a pull in force indicating a change from a repulsive to an attractive system.

In detail, Figure 4a shows the *F*–*D* curve for a compression at −0.2 V with a switch to +0.7 V. Here, the black points show the expected hydration repulsion during approach at negative potential. Upon switching polarization, a slope change in the compression curve (red points) is evident. Because of expulsion of adsorbed cations, the surfaces are pulled closer together. Upon separation the adhesion force of *F*_adh_ = −4.6 mN/m compares well with the equilibrium data for +0.7 V shown in Figure 2c.

In Figure 4b, when reversing polarization during compression at +0.7 V and subsequent separation at −0.2 V, no outward shift is observed. The separation curve (black points) follows the same path as the compression with minimal adhesion as expected for −0.2 V. Interestingly, there is no significant outward shift toward the expected equilibrium curve, indicating that cations appear to not be able to enter the contact zone under the applied load. This is an interesting result indicative of a hysteresis of charge regulation, with cations being able to exit a repolarizing contact zone of a moderately compressed bilayer at about 10 mN/m. However, reentering is considerably hindered by the contact pressure.

In summary, at low ionic strength the DLVO model and, specifically, potential dependent Gouy–Chapman double layer forces describe interaction force profiles well. At high ionic strength the surface charging of a bilayer is screened entirely within the hydration layer, which is effectively an inner Helmholtz layer. We further demonstrated a reversible switch between repulsive and attractive interaction due to adsorption and desorption of Na^+^ primarily on the headgroups of the bilayer via variation of potential dependent steric hydration forces. This suggests that the DLVO model is not at all applicable to physiologic conditions and bilayer interactions. In contrast, exponential hydration layer interactions effectively describe the interaction forces in an extended DLVO approach, experimentally proving the importance of steric hydration effects on bilayer–bilayer interactions. By extension this experimental approach could also be applied to bilayer–substrate interactions. Furthermore, these data confirm that steric hydration forces originate from ion adsorption at an interface. Our approach will prove useful for systematically unravelling the exponential nature of the hydration layer forces and their dependence on surface potential variations, which may act as biological triggers for function or signaling.

The raw and processed data required to reproduce these findings are available from the corresponding author via www.repositum.tuwien.ac.at upon reasonable request, and the neutron reflectometry data are archived under doi:10.5291/ILL-DATA.9-11-2022.

## Supporting Information Available

Experimental details, materials, and methods; additional neutron reflectivity data and calculation for extended DLVO fitting (PDF)
