# Supplementary material for: Hydration Forces Dominate Surface Charge Dependent Lipid Bilayer Interactions under Physiological Conditions
Source: J Phys Chem Lett. 2021 Sep 17;12(38):9248–52. doi: 10.1021/acs.jpclett.1c02572 (PMC8488952; doi:10.1021/acs.jpclett.1c02572)
Supplement: Supplementary file 1 — jz1c02572_si_001.pdf [file jz1c02572_si_001.pdf]

Supporting Information
Hydration Forces Dominate Surface Charge Dependent Lipid Bilayer Interactions under Physiological Conditions

Valentina Wieser,† Laura L. E. Mears,† Robert D. Barker,‡ Hsiu-Wei Cheng,† and Markus Valtiner*,†

†*Vienna University of Technology, Institute for Applied Physics, Wiedner Hauptstrasse 8-10, A-1040 Vienna, Austria*

‡*School of Physical Sciences, University of Kent, Canterbury, United Kingdom*

E-mail: markus.valtiner@tuwien.ac.at*

S1. CHEMICALS AND MATERIALS

The tethered bilayer lipid membrane system consists of an inner layer of 2,3-di-O-phytanyl-sn-glycerol-1-tetraethyleneglyco-D, L-α-lipoic acid ester (DPhyTL, custom made from Celestial Synthetics, Australia) stored in HPLC-grad Ethanol (VWR). The outer lipid layer is comprised of 1,2-dioleoyl-sn-glycero-3-phosphocholine (DOPC) stored in chloroform (99.9 %, Sigma Aldrich). SFA experiments were conducted in sodium chloride (99.9%, Sigma Aldrich) solution. Salt solutions were prepared with Milli-Q water (Merck), or D_2O (99.9%, Sigma Aldrich) where noted.

S2. ELECTROCHEMICAL 3-MIRROR INTERFEROMETER SURFACE FORCES APPARATUS

We show results obtained with a home built Surface Forces Apparatus equipped with an electrochemical cell and adapted for a 3-mirror interferometer geometry. The set up is introduced in detail in our previous work.[S1] Briefly, the 3-mirror interferometer SFA geometry consists of two cross-cylindrical gold surfaces prepared in a multi layer process. The gold surfaces function on one hand as a substrate for stable tBLMs and on the other hand as working electrodes (W.E.) for symmetric electrochemical modulation of the bilayers. After gluing, via UV cured glue, of back-silvered mica onto the cylindrical quartz glass disk with radius of curvature = 1 cm, a thin gold wire (Goodfellow) is glued to the side of the disk for electrical connection (see Fig.1 a)-b)). After a second UV curing process a thin titanium adhesion layer is sputtered onto the mica/wire (40 W, Argon background pressure = 10^{-2} mbar). Afterwards, we evaporate a 10 nm gold film onto the titanium adhesion layer (0.1 Å/s, background pressure = 10^{-6} mbar). The apposing gold surface consists of a 40-50 nm thick template stripped gold thin film[S2] with a gold wire pressed onto the edge of the gold disk. A platinum wire bent into a hook around the surfaces functions as counter electrode (C.E) and a mini Ag/AgCl electrode is used as reference electrode (R.E). The final set up with the surfaces and wire connections is shown in Fig.1. Analysis of the measured interferometric data for separation distance calculation was carried out with the SFA Explorer software package

for 3-mirror configuration.[S3]

S3. TETHERED BILAYER LIPID MEMBRANES

In order to perform force-distance (F-D) measurements on stable bilayer systems we employ symmetric functionalization of the gold surfaces with tethered bilayer lipid membranes.[S4] Using tBLMs on gold substrates makes stable, reproducible measurements possible, which is otherwise not given for bilayers on substrates like e.g. mica as lipids are only weakly, electrostatically bound to the substrate.[S5] We functionalize the gold surfaces first with a monolayer of DPhyTL which covalently binds to the gold via a thiol-gold bond during a self assembly process. The gold surfaces are incubated for 1 hour in a 0.1 mg/ml ethanolic solution. To remove any unbound excess molecules after the incubation we rinse the surfaces thoroughly with ethanol and dry them gently under a N_2 stream. The outer lipid leaflet, consisting of DOPC, is deposited in a Langmuir Blodgett trough at a surface pressure of Π = 40 mN/m, equal to an area per molecule of 80Å^2. For that 300 μl of a 0.1 mg/ml DOPC-chloroform mixture is dispersed onto the Milli-Q water surface of the LBT. After deposition of the outer DOPC leaflet the cell is assembled in the Milli-Q water and transferred to the SFA.

S4.NEUTRON REFLECTOMETRY

Measurements were carried out on the D17 vertical reflectometer at the Institut Laue-Langevin, Grenoble, France.[S6,S7] We operated the instrument in time of flight mode collecting data at two angles (1.2° and 4.2°) to achieve the required range in Q to record multiple fringes. The tethered DPhyTL and Ti adhered 10 nm gold layers were prepared as above onto polished silicon blocks of 10 mm thickness and 75 mm diameter. The blocks were clamped to a PEEK trough to form a solid-liquid cell which was connected to a HPLC pump for solution exchange. Full exchange was ensured by pumping more than ten times the volume of the cell through before commencing the next measurement. The data from the two angles were reduced and combined using the COSMOS

reduction software.[S8]

S5. DLVO AND HYDRATION REPULSION FITTING

Molecular interaction forces between solids in liquid are well described by Derjaguin–Landau–Verwey–Overbeek theory for cross-cylindrical geometry, which includes electric double layer and van der Waals forces. However, interaction between biologic systems such as bilayers are not fully described with this theory as it does not account for any steric hydration effects on the hydrophilic headgroups. Therefore the bilayer interaction and force modulation can only be explained with the addition of a hydration force term encapsulating the repulsive force arising from water and ion structuring on the interface.[S9,S10]

$$\begin{aligned}\frac{F}{R} = -\frac{A_{H,bilayer}}{6(D_0-D_{HW})^2} - \frac{A_{H,Au}}{6(D_0-D_{Au})^2} + \frac{A_{H,Au-medium}}{6(D_0-D_{Au})^2} \\ +\kappa(9.22*10^{-11})tanh^2(\frac{\psi}{103})e^{-\kappa(D_0-D_{HW})} \\ +W_0 e^{-\frac{(D_0-D_{HW})}{\lambda_{hydra}}}\end{aligned} \quad (1)$$

Here, the first three terms describe the van der Waals (vdW) forces of the multilayer system comprised of interaction between the bilayers, between the gold substrates and the gold with the electrolyte medium, respectively.[S11,S12] $A_{H,bilayer}$ describes the Hamaker constant for bilayers, which is estimated as 10^{-20} J. The Hamaker constant is slightly higher than what would be normally assumed for lipid bilayers[S10,S12] due to the denser tethered system incorporating both lipid layers as well as hydrocarbon chains.[S9] $A_{H,Au}$ is the Hamaker constant for gold-gold interaction and $A_{H,Au-medium}$ accounts for the Hamaker constant for the interaction between the gold and surrounding electrolyte medium and is estimated at $5 \cdot 10^{-20}$ J. The influence of this parameter is however negligible for the overall fit, and cannot be precisely estimated. D_0 is the separation distance between bilayer head groups, referenced to the contact hard wall ($D_0 = 0$) of the bilayers in Milli-Q water. The experimental reference for the DOPC layer thickness was taken from a contact

between DPhyTL coated gold surfaces to avoid cold welding between the gold. D_{HW} and D_{Au} are the hard wall distances referring to the minimal distance at which the vdW forces are acting. Whereas D_{Au} stays constant over the experiments, D_{HW} is shifted in high salt concentrations as the plane of origin for the vdW force between the bilayers is congruent with the DOPC thickness which increases due to swelling. The fourth term describes the electric double layer repulsion for symmetric surfaces after the Gouy-Chapman model where κ^{-1} is the Debye length, ψ is the surface potential and D_{HW} is the hard wall distance for the minimal compression distance for EDL forces. The last term describes the hydration force fitted with a simple exponential decay. Here, W_0 is the hydration constant, λ_{hydra} is the hydration decay length and D_{HW} is again the hard wall distance for the hydration force.

SUPPLEMENTARY DATA

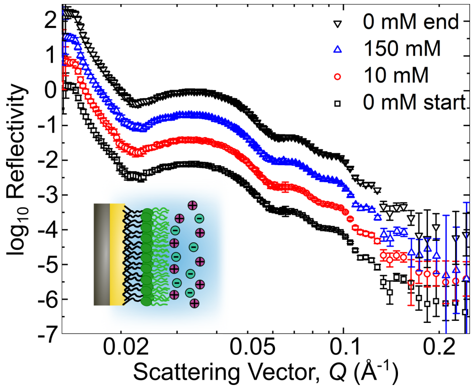

Figure S1: Neutron reflectometry profiles of the DPhyTL support layer tethered to a gold thin film on top of a silicon block as indicated in the inset. The reflectivity was measured from the buried interface against D_2O with salt concentrations 10 and 150 mM and without salt before (0 mM start) and after (0 mM end) the measurements with salt. No differences in layer thickness are visible between the measurements. The data sets have been offset vertically for clarity.

References

(S1) Wieser, V.; Bilotto, P.; Ramach, U.; Yuan, H.; Schwenzfeier, K.; Cheng, H.-W.; Valtiner, M. Novel in situ sensing surface forces apparatus for measuring gold versus gold, hydrophobic, and biophysical interactions. *Journal of Vacuum Science & Technology A: Vacuum, Surfaces, and Films* **2021**, *39*, 023201.

(S2) Valtiner, M.; Banquy, X.; Kristiansen, K.; Greene, G. W.; Israelachvili, J. N. The electrochemical surface forces apparatus: The effect of surface roughness, electrostatic surface potentials, and anodic oxide growth on interaction forces, and friction between dissimilar surfaces in aqueous solutions. *Langmuir* **2012**, *28*, 13080–13093.

(S3) Schwenzfeier, K. A.; Erbe, A.; Bilotto, P.; Lengauer, M.; Merola, C.; Cheng, H.-W.; Mears, L. L.; Valtiner, M. Optimizing multiple beam interferometry in the surface forces apparatus: Novel optics, reflection mode modeling, metal layer thicknesses, birefringence, and rotation of anisotropic layers. *Review of Scientific Instruments* **2019**, *90*, 043908.

(S4) Andersson, J.; Köper, I. Tethered and polymer supported bilayer lipid membranes: Structure and function. *Membranes* **2016**, *6*, 30.

(S5) Bilotto, P.; Lengauer, M.; Andersson, J.; Ramach, U.; Mears, L. L.; Valtiner, M. Interaction Profiles and Stability of Rigid and Polymer-Tethered Lipid Bilayer Models at Highly Charged and Highly Adhesive Contacts. *Langmuir* **2019**, *35*, 15552–15563.

(S6) Cubitt, R.; Fragneto, G. D17 : the new reflectometer at the ILL. *Applied Physics A, Materials Science and Processing* **2002**, *74*, S329–S331.

(S7) Saerbeck, T.; Cubitt, R.; Wildes, A.; Manzin, G.; Andersen, K. H.; Gutfreund, P. Recent upgrades of the neutron reflectometer D17 at ILL. *Journal of Applied Crystallography* **2018**, *51*, 249–256.

(S8) Gutfreund, P.; Saerbeck, T.; Gonzalez, M. A.; Pellegrini, E.; Laver, M.; Dewhurst, C.; Cubitt, R. Towards generalized data reduction on a chopper-based time-of-flight neutron reflectometer. *Journal of Applied Crystallography* **2018**, *51*, 606–615.

(S9) Israelachvili, J. N. *Intermolecular and surface forces*; Academic press, 2015.

(S10) Leckband, D.; Israelachvili, J. Intermolecular forces in biology. *Quarterly reviews of biophysics* **2001**, *34*, 105.

(S11) Parsegian, V. A. *Van der Waals forces: a handbook for biologists, chemists, engineers, and physicists*; Cambridge University Press, 2005.

(S12) Anderson, T. H.; Donaldson, S. H.; Zeng, H.; Israelachvili, J. N. Direct measurement of double-layer, van der Waals, and polymer depletion attraction forces between supported cationic bilayers. *Langmuir* **2010**, *26*, 14458–14465.
